# Sex differences in the clinical manifestation of autosomal dominant frontotemporal dementia

**DOI:** 10.1002/alz.14630

**Published:** 2025-04-25

**Authors:** Molly Memel, Adam M. Staffaroni, Ignacio Ilan‐Gala, Jesús Garcia Castro, John Kornak, Carmela M. Tartaglia, Rowan Saloner, Anna M. VandeBunte, Emily W. Paolillo, Claire J. Cadwallader, Coty Chen, Maria Luisa Gorno‐Tempini, Malu Mandelli, Liana Apostolova, Neil Graff‐Radford, Irene Litvan, Ece Bayram, Peter S. Pressman, Toji Miyagawa, Ian Mackenzie, Jill Goldman, Richard R. Darby, Brian S. Appleby, Len Petrucelli, Tania Gendron, Hilary W. Heuer, Leah K. Forseberg, Julio C. Rojas, Brad F. Boeve, Nellie Brushaber, Kimiko Domoto‐Reilly, Nupur Ghoshal, Maria Lapid, Belen Pascual, Suzee Lee, Eliana Marisa Ramos, Vijay Ramanan, Rosa Rademakers, Katya Rascovsky, Alex Pantelyat, Joseph C. Masdeu, Allison Snyder, Adam L Boxer, Howard J. Rosen, Kaitlin Casaletto, the ALLFTD Consortium

**Affiliations:** ^1^ Ray Dolby Brain Health Center Sutter Health San Francisco California USA; ^2^ Memory and Aging Center, Department of Neurology University of California San Francisco California USA; ^3^ Neurology, Sant Pau Memory Unit Hospital de la Santa Creu i Sant Pau Barcelona Spain; ^4^ Epidemiology & Biostatistics University of California San Francisco School of Medicine San Francisco California USA; ^5^ Department of Medicine University of Toronto Toronto Ontario Canada; ^6^ Department of Neurology Indiana University School of Medicine Indianapolis Indiana USA; ^7^ Department of Neurology Mayo Clinic Jacksonville Florida USA; ^8^ Department of Neurosciences University of California San Diego La Jolla California USA; ^9^ Department of Neurology University of Colorado School of Medicine Aurora Colorado USA; ^10^ Mayo Clinic Rochester Minnesota USA; ^11^ Pathology and Laboratory Medicine The University of British Columbia Vancouver British Columbia Canada; ^12^ The Neurological Institute – Columbia University Irving Medical Center New York New York USA; ^13^ Department of Neurology Vanderbilt University Nashville Tennessee USA; ^14^ Department of Neuroscience Mayo Clinic Jacksonville Florida USA; ^15^ Department of Neurology Mayo Clinic Rochester Minnesota USA; ^16^ Quantitative Health Sciences Mayo Clinic Rochester Minnesota USA; ^17^ Department of Neurology University of Washington Seattle Washington USA; ^18^ Department of Neurology Washington University in St. Louis School of Medicine St. Louis Missouri USA; ^19^ Houston Methodist Houston Texas USA; ^20^ David Geffen School of Medicine at UCLA Los Angeles California USA; ^21^ University of Antwerp Antwerpen Belgium; ^22^ Department of Neurology and Neuropsychology University of Pennsylvania Philadelphia Pennsylvania USA; ^23^ Department of Neurology Johns Hopkins University School of Medicine Baltimore Maryland USA; ^24^ National Institutes of Health Bethesda Maryland USA

**Keywords:** cognitive resilience, frontotemporal dementia, neurofilament light chain, sex

## Abstract

**INTRODUCTION:**

Sex differences are apparent in neurodegenerative diseases but have not been comprehensively characterized in frontotemporal dementia (FTD).

**METHODS:**

Participants included 337 adults with autosomal dominant FTD enrolled in the ALLFTD Consortium. Clinical assessments and plasma were collected annually for up to 6 years. Linear mixed‐effects models investigated how sex and disease stage are associated with longitudinal trajectories of cognition, function, and neurofilament light chain (NfL).

**RESULTS:**

While sex differences were not apparent at asymptomatic stages, females showed more rapid declines across all outcomes in symptomatic stages compared to males. In asymptomatic participants, the association between baseline NfL and clinical trajectories was weaker in females versus males, a difference that was not present in symptomatic participants.

**DISCUSSION:**

In genetic FTD, females show cognitive resilience in early disease stages followed by steeper clinical declines later in the disease. Baseline NfL may be a less sensitive prognostic tool for clinical progression in females with FTD‐causing mutations.

**Highlights:**

- Females with genetic FTD exhibit overall steeper increases in plasma neurofilament light chain (NfL) than males.
- Females with genetic FTD outperform NfL levels in asymptomatic stages compared to males.
- Once symptomatic, females with genetic FTD decline more rapidly than males.
- Plasma NfL is a stronger prognostic marker in asymptomatic males than females.

## 1 BACKGROUND

Females are disproportionately affected by dementia compared to males.^1^ In Alzheimer's disease (AD), asymptomatic amyloid‐positive females show better clinical functioning despite comparable hippocampal atrophy and temporal lobe glucose metabolism,^2^ and greater tau burden^3^ compared to males; yet, they show a hastened rate of cognitive decline,^4^, ^5^ brain atrophy,^6^, ^7^ and functional progression once becoming symptomatic.^5^ Similar patterns are observed in genetic and sporadic forms of early onset AD (presentation before age 65) as well. In individuals with mild cognitive impairment and mild dementia due to early onset AD, females exhibit significantly higher cerebrospinal fluid (CSF) total tau and phosphorylated tau levels than males in the setting of similar cognitive abilities,^8^ and females with autosomal dominant forms of AD also showed a steeper increase in plasma neurofilament light chain (NfL) with disease progression (proxied by age).^9^ Despite being a leading cause of early age of dementia onset, little is known about sex differences in the presentation and clinical course of frontotemporal dementia spectrum disorders (FTD). Understanding the unique clinical presentation of neurodegenerative diseases by sex is a fundamental step toward accurate diagnosis and patient care. Further, identifying sex‐specific biological vulnerabilities will inform biomarker and therapy targets in support of ongoing efforts for precision dementia care in people living with FTD.

Few studies have examined sex differences in FTD, particularly in large cohorts with longitudinal follow‐up. Among sporadic cases of FTD, behavioral variant FTD (bvFTD) may be more prevalent in males,^10^ whereas primary progressive aphasia may be more prevalent among females in some cohorts,^11^ but not others.^10^ Sex differences in clinical phenotype are not typically found among genetic/familial cases of FTD.^10^ Regarding clinical outcomes, one cross‐sectional study including sporadic and autosomal dominant FTD cases demonstrated a later age of symptom onset in females; worse memory, executive functions, and visuoconstructional abilities in females; and worse psychiatric symptoms in males.^11^ Another study focused exclusively on individuals with bvFTD demonstrated greater atrophy at diagnosis in females than males despite better than expected executive and psychiatric functioning based on their degree of neurodegeneration.^12^ Together, these cross‐sectional studies suggest that there are important sex differences in FTD, potentially characterized as “cognitive resilience” against neurodegenerative changes in females—at least in the early stages of the disease continuum—but potentially worse cognitive outcomes later in disease.

There is a lack of existing work comprehensively characterizing how sex may impact the longitudinal clinical manifestation of disease in FTD. FTD is unique in that more than one third of cases are familial with ≈ 20% of cases estimated to be attributable to one of three genes bearing autosomal dominant mutations (*C9orf72, GRN*, or *MAPT*). Evaluating sex differences in FTD‐causing autosomal dominant mutations allows for a comprehensive modeling of the natural history of the disease, especially at asymptomatic stages. In this study, we examined sex differences in longitudinal clinical trajectories of 373 participants with autosomal dominant forms of FTD enrolled in the ARTFL‐LEFFTDS Longitudinal Frontotemporal Lobar Degeneration (ALLFTD) Consortium. Specifically, we examined sex differences in longitudinal trajectories of cognition, function, and plasma NfL concentration, one of the most robust prognostic biomarkers for frontotemporal lobar degeneration (FTLD).^13^, ^14^, ^15^, ^16^ Based on prior work, we hypothesized that females would exhibit better clinical outcomes than males given their NfL concentration, suggestive of possible “cognitive resilience.” These data support efforts toward more precise dementia care with specific attention to previously understudied aspects of women's brain health that will be critical to consider as novel biomarkers and therapies are developed for FTD.

## 2 METHODS

### 2.1 Participants

Participants were recruited through the ALLFTD Study, which is a consortium of 28 centers across the United States and Canada focused on identifying individuals and family members of those with FTD. We included all participants who were carriers of pathogenic variants in the three most common genes in autosomal dominant FTD (*MAPT*, *GRN*, and *C9orf72*; Table 1).

**TABLE 1:** Participant demographics at baseline—means and standard deviations.

	Males (*N* = 169)	Females (*N* = 204)	*t* statistic/chi‐square	*p*‐value
Age (years)	50.3 (14.3)	50.9 (14.6)	−0.39	0.70
Education (years)	15.5 (2.5)	17.5 (2.1)	0.60	0.55
Race (% White)	163 (96%)	199 (98%)		
Pathogenic variant			2.56	0.28
*MAPT*	43 (25.4%)	56 (27.5%)		
*GRN*	44 (26.0%)	39 (19.1%)		
*C9orf72*	82 (48.5%)	109 (53.4%)		
Baseline disease stage (*N*)			4.18	0.38
Asymptomatic	82 (49%)	106 (52%)		
MBCI	30 (18%)	29 (14%)		
Dementia	57 (34%)	69 (34%)		
CDR + NACC FTLD SB	3.2 (4.6)	4.2 (6.4)	−1.74	0.08
Disease stage converters	24 (14%)	14 (7%)	0.51	0.48
Study informant sex				
Male	%	87%	149.12	0.000
Female	99%	13%		
Global cognition	0.3 (0.6)	0.5 (0.5)	−1.85	0.07
MoCA	24.0 (7.8)	25.6 (13.2)	−1.31	0.19
Plasma neurofilament light chain (pg/mL)^*^	17.2 (18.4)	25.2 (35.0)	−2.46	0.01
Total number of study visits	2.4 (1.5)	2.4 (1.4)	0.22	0.83
Inter‐visit interval	0.7 (0.7)	0.7 (0.8)	−0.32	0.75

Abbreviations: CDR + NACC FTLD SB, Clinical Dementia Rating dementia staging instrument plus National Alzheimer's Coordinating Center behavior and language domains frontotemporal lobar degeneration module sum of boxes; MoCA, Montreal Cognitive Assessment.

* Statistically significant at *P* = 0.01.

### 2.2 Clinical assessment

The ALLFTD protocol includes annual evaluations allowing for longitudinal assessment of clinical and functional trajectories. The multidisciplinary assessment includes neurological examination, informant interview, and neuropsychological assessment. Cognitive testing included the third version of the National Institute on Aging (NIA) Alzheimer's Disease Centers’ Uniform Dataset Neuropsychological Test Battery (UDSv3),^17^, ^18^ supplemented with a UDS module for assessment of FTD. We used a metric of global cognition based on performance across nine measures of memory, language, and executive functioning. To create the composite metric, sample‐specific *z* scores were first calculated for each of the individual tasks (based on all time points available for all participants), averaged within cognitive domains, and then averaged across domains.

**Research in context**

1. **Systematic review**: The authors performed a literature review on sex differences in the clinical course of frontotemporal dementia (FTD) using PubMed. Prior work has characterized sex differences in the cognitive and behavioral phenotype of FTD. Less is known about longitudinal cognitive and functional trajectories, particularly among individuals with familial FTD.
2. **Interpretation**: Females show steeper neurofilament light chain (NfL) increases overall, yet outperform NfL levels at asymptomaptic stages compared to males. Once becoming symptomatic, females exhibit more precipitous declines than males. Our findings suggest cognitive resilience in females with autosomal dominant FTD and highlight limitations in the utility of NfL as a prognostic marker in asymptomatic females.
3. **Future directions**: Larger sample sizes are needed to determine sex differences among specific variants of genetic FTD. Additionally, it will be important to determine whether sex differences in clinical phenotype at symptom onset account for the apparent delay in age at diagnosis and the course of clinical decline in females.

Functional status was measured using the Clinical Dementia Rating (CDR) scale plus Behavioral and Language Domains from the National Alzheimer's Coordinating Center (NACC) FTLD module (CDR+NACC FTLD).^19^, ^20^ Two different metrics from the CDR+NACC FTLD were used. The CDR+NACC FTLD Global Score (interval) was based on eight functional domain scores (memory, orientation, judgment/problem solving, community affairs, home and hobbies, personal care, language, and behavior) and used as a predictor with participants grouped into asymptomatic (CDR+NACC FTLD = 0), mild behavioral and/or cognitive impairment (MBCI^21^; CDR+NACC FTLD = 0.5), and dementia (CDR+NACC FTLD > 0.5). The second metric is the CDR+NACC FTLD sum of boxes (CDR+NACC FTLD‐SB), which is the total score across the aforementioned domains of cognitive and functional performance. The CDR+NACC FTLD‐SB score was recorded as a semi‐continuous outcome variable (range 0–24, higher scores indicate more impairment).

### 2.3 Genetic screening

Participants had genetic testing performed in the same laboratory at University of California Los Angeles using previously published methods^22^ and were included if they were carriers of pathogenic variants in one of the three most common genes in autosomal dominant FTD (*MAPT*, *GRN*, and *C9orf72*).^23^

### 2.4 Neurofilament Light Chain (NfL)

Plasma NfL was acquired through a collection of venous blood as previously described.^24^

### 2.5 Statistical analyses

Baseline sex differences in demographic and clinical variables were analyzed via independent sample *t* tests and chi‐square tests, as appropriate. Linear mixed effects (LME) models investigated sex differences in cognitive, functional (CDR+NACC FTLD‐SB), and plasma NfL trajectories. All models estimated subject‐specific intercepts and slopes and adjusted for baseline age and education. We first examined two‐way interactions between sex and time in study (years) on outcome variables. Given that sex differences have been found to emerge by disease stage in other neurodegenerative diseases,^4^, ^5^, ^25^ we next investigated the interaction among sex, baseline disease stage (i.e. asymptomatic, MBCI, dementia), and time on neurobehavioral outcomes. To enhance interpretability by reducing the number of interaction terms to be considered, each combination of sex (male, female) and disease stage group (normal, MBCI, dementia) was considered a separate level within a combined categorical predictor. Asymptomatic men were used as the reference group for tables to understand female‐specific trajectories, and additional pairwise comparisons of interest using different reference groups are reported in the text. Primary models included raw NfL values as the outcome, given we observed approximately normally distributed residuals from these model fits.

Finally, given the role of NfL as a prognostic biomarker in FTD,^24^ we investigated how the relationship between baseline plasma NfL and clinical outcomes differed between males and females (sex x baseline NfL x time). Further, to determine whether the latter effects varied by disease stage, we stratified models by baseline disease stage. Unstandardized regression coefficients (*b*) and standard errors (SE) are reported. Statistically significant interactions (*P* < 0.05) were probed by plotting mean slopes conditional on low and high values (25th and 75th percentile) of continuous predictors (e.g., baseline NfL levels) derived from the entire sample.

Due to differences in the rate of neurodegeneration and clinical progression among genetic subtypes of FTD,^24^, ^26^ exploratory post hoc analyses investigated whether findings differed across pathogenic variants (*MAPT, GRN, C9orf72*). Further, sensitivity analyses examined whether removing patients with amyotrophic lateral sclerosis (ALS) and FTD/ALS changed the primary results, as ALS impacts a wider network of both peripheral and central neuronal pathways with correspondingly high peripheral NfL signal.

## 3 RESULTS

### 3.1 Baseline sex differences

At baseline, females and males did not statistically differ on genotype, demographic, cognitive, or functional variables (see Table 1). Females with MBCI at baseline were more likely to exhibit disinhibition than males with MBCI (Table S1 in supporting information), whereas the reverse was true in participants with dementia at baseline; no other behavioral symptoms differed by sex. Although there were no statistically significant sex differences in baseline age at each disease stage, the average age of females in the MBCI group was 5 years older than males (51 vs. 56 years; mean difference = 5.4, 95% confidence interval [CI: –11.9, 1.0]); versus asymptomatic (43 vs. 43 years; mean difference = 0.3, 95% CI [–3.7, 4.2]) and dementia (60 vs. 61 years; mean difference = 0.6, 95% CI [–3.9, 2.6]; see Table S1). Female pathogenic variant carriers also had a higher plasma NfL concentration overall (17.2 vs. 25.2 pg/mL, *P* = 0.014). This difference was primarily present in individuals with dementia at baseline. Females with dementia showed higher baseline NfL compared to males (mean = 49.0 pg/mL vs. 29.4 pg/mL, mean difference = 19.4, 95% CI [–33.0, –5.7], *P* = 0.006; see Table S1), whereas participants who were asymptomatic or MBCI at baseline did not differ on NfL concentrations (asymptomatic: 8.2 pg/mL vs. 9.2 pg/mL, *t*[156] = –0.46, *P* = 0.650; MBCI: 18.0 pg/mL vs. 22.3 pg/mL, *t*(47) = –0.74, *P* = 0.466).

### 3.2 Sex differences in cognitive, functional, and NfL trajectories

Overall, females and males did not statistically differ on longitudinal trajectories of cognition or functional severity (CDR+NACC FTLD‐SB; Table S2 in supporting information). However, females exhibited a more rapid increase in plasma NfL over the study than males (9.2 pg/mL annual increase in females; 1.3 pg/mL annual increase in males). When removing one possibly outlying datapoint with very elevated NfL (326 pg/mL) from the analysis, the interaction between sex and time on NfL remained significant (*b* = 2.04, SE = 0.91, *P* = 0.026, 95% CI [0.25, 3.83]). Removing participants with ALS and FTD/ALS, a group for whom more extensive neuronal damage may occur, attenuated the effect such that it became marginal though the effect size was similar (*b* = 3.67, SE = 1.94, *P* = 0.058, 95% CI [–0.12, 7.50]).

**FIGURE 1:**
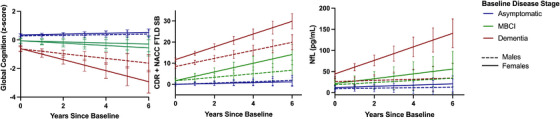
Female FTD pathogenic variant carriers exhibit more rapid clinical trajectories after symptom manifestation. Predicted plots based on linear mixed effects models, bars represent 95% confidence interval. CDR + NACC FTLD, Clinical Dementia Rating dementia staging instrument plus National Alzheimer's Coordinating Center behavior and language domains frontotemporal lobar degeneration module; FTD, frontotemporal dementia; MBCI, mild behavioral/cognitive impairment; NfL, neurofilament light chain.

When modeling the influence of sex and baseline disease stage at study entry (asymptomatic, MBCI, dementia) using the six‐level combined variable, there were statistically significant group differences across longitudinal cognitive, functional, and NfL trajectories (Table 2; Figure 1). Across clinical outcomes, females and males who were asymptomatic at baseline did not differ on cognitive, functional, or NfL trajectories (see Table 2 and Figure 1). In contrast, females with MBCI at baseline exhibited steeper declines in cognition and function than males who were asymptomatic (cognition: *b* = 0.10, SE = 0.05, *P* = 0.024, 95% CI [0.01, 0.20]) or with MBCI (function: *b* = –1.23, SE = 0.57, *P* = 0.030, 95% CI [–0.12, –2.34]), but not compared to males with dementia (cognition: *b* = –0.07, SE = 0.06, *P* = 0.286, 95% CI [–0.19, 0.06], function: *b* = 0.15, SE = 0.50, *P* = 0.763, 95% CI [–0.84, 1.14]). Females with MBCI exhibited a slower increase in NfL than females with dementia (*b* = 10.31, SE = 4.56, *P* = 0.024, 95% CI [1.38, 19.25]), but did not differ from other groups in the rate of NfL change over time. Females with dementia at baseline exhibited the steepest declines in cognition and function and greatest increases in NfL concentrations compared to all other groups. Upon removing the aforementioned NfL outlier, the interaction on NfL trajectories remained the same. Similarly, NfL trajectories were unchanged when excluding participants with FTD/ALS.

**TABLE 2:** Linear mixed effects models demonstrating disease stage at study entry moderates sex differences on longitudinal cognitive, functional, and plasma neurofilament light chain (NfL) trajectories.

	Global cognition		CDR + NACC FTLD SB		Plasma NfL	
	Unstandardized effect size (SE)	95% CI	Unstandardized effect size (SE)	95% CI	Unstandardized effect size (SE)	95% CI
Baseline age	−0.01 (0.00)^***^	[–0.01, 0.00]	−0.01 (0.00)^***^	[–0.01, 0.00]	0.29 (0.11)^*^	[0.07, 0.52]
Education	0.05 (0.01)^***^	[0.03, 0.07]	−0.05 (0.01)^***^	[0.03, 0.07]	0.91 (0.56)	[–0.20, 2.01]
Group						
Males with MBCI	−0.34 (0.10)^***^	[–0.54, –0.15]	1.69 (0.68)^*^	[0.36, 3.03]	9.44 (5.80)	[–1.93, 20.81]
Males with dementia	−0.91 (0.10)^***^	[–1.10, –0.72]	8.45 (0.59)^***^	[7.29, 9.62]	16.78 (5.07)^***^	[6.84, 26.73]
Asymptomatic females	0.07 (0.07)	[–0.07, 0.20]	0.00 (0.46)	[–0.90, 0.90]	2.65 (3.96)	[–5.11, 10.42]
Females with MBCI	−0.34 (0.11)^**^	[–0.55, –0.13]	1.75 (0.70)^*^	[0.37, 3.12]	12.20 (6.31)	[–0.16, 24.55]
Females with dementia	−0.89 (0.12)^***^	[–1.11, –0.66]	11.70 (0.57)^***^	[10.58, 12.82]	34.96 (4.83)^***^	[25.48, 44.43]
Time since baseline	0	[–0.01, 0.07]	0.31 (0.20)	[–0.09, 0.71]	0.59 (1.63)	[–2.60, 3.79]
Group x time						
Males with MBCI	−0.06 (0.05)	[–0.15, 0.04]	0.51 (0.45)	[–0.37, 1.39]	1.94 (3.39)	[–4.70, 8.59]
Males with dementia	−0.19 (0.05)^**^	[–0.30, –0.08]	1.59 (0.37)^***^	[0.87, 2.31]	0.78 (3.36)	[–5.81, 7.38]
Asymptomatic females	0.01 (0.03)	[–0.05, 0.07]	−0.12 (0.27)	[–0.65, 0.41]	0.86 (2.25)	[–3.55, 5.28]
Females with MBCI	−0.11 (0.05)^*^	[–0.20, –0.01]	1.74 (0.45)^***^	[0.86, 2.63]]	5.11 (3.88)	[–2.50, 12.72]
Females with dementia	−0.41 (0.07)^***^	[–0.55, –0.27]	2.71 (0.37)^***^	[1.99, 3.43]	15.42 (3.32)^***^	[8.91, 21.93]

*Note*: Reference group: asymptomatic males.

Abbreviations: CDR + NACC FTLD SB,  Clinical Dementia Rating dementia staging instrument plus National Alzheimer's Coordinating Center behavior and language domains frontotemporal lobar degeneration module sum of boxes; CI, confidence interval; MBCI, mild behavioral/cognitive impairment; NfL, neurofilament light chain; SE, standard error.

* *P* < 0.05.

** *P* < 0.01.

*** *P* ≤ 0.001.

Given potential biases in the degree of symptom endorsement in male versus female informants, sensitivity analyses were conducted controlling for informant sex in the above analyses. Notably, the gender of the informant was not known in 29.4% of the sample, which significantly reduced the sample size. Nonetheless, controlling for informant sex did not change the similar clinical, functional, and NfL trajectories in asymptomatic participants, and the pattern of steepest clinical and functional trajectories in females with dementia compared to all other groups. The impact of informant sex on the age at diagnosis and degree of cognitive, behavioral, and functional decline endorsed is an important consideration in FTD and warrants further study as discussed below.

### 3.3 Sex differences in the relationship between baseline NfL and clinical trajectories

**FIGURE 2:**
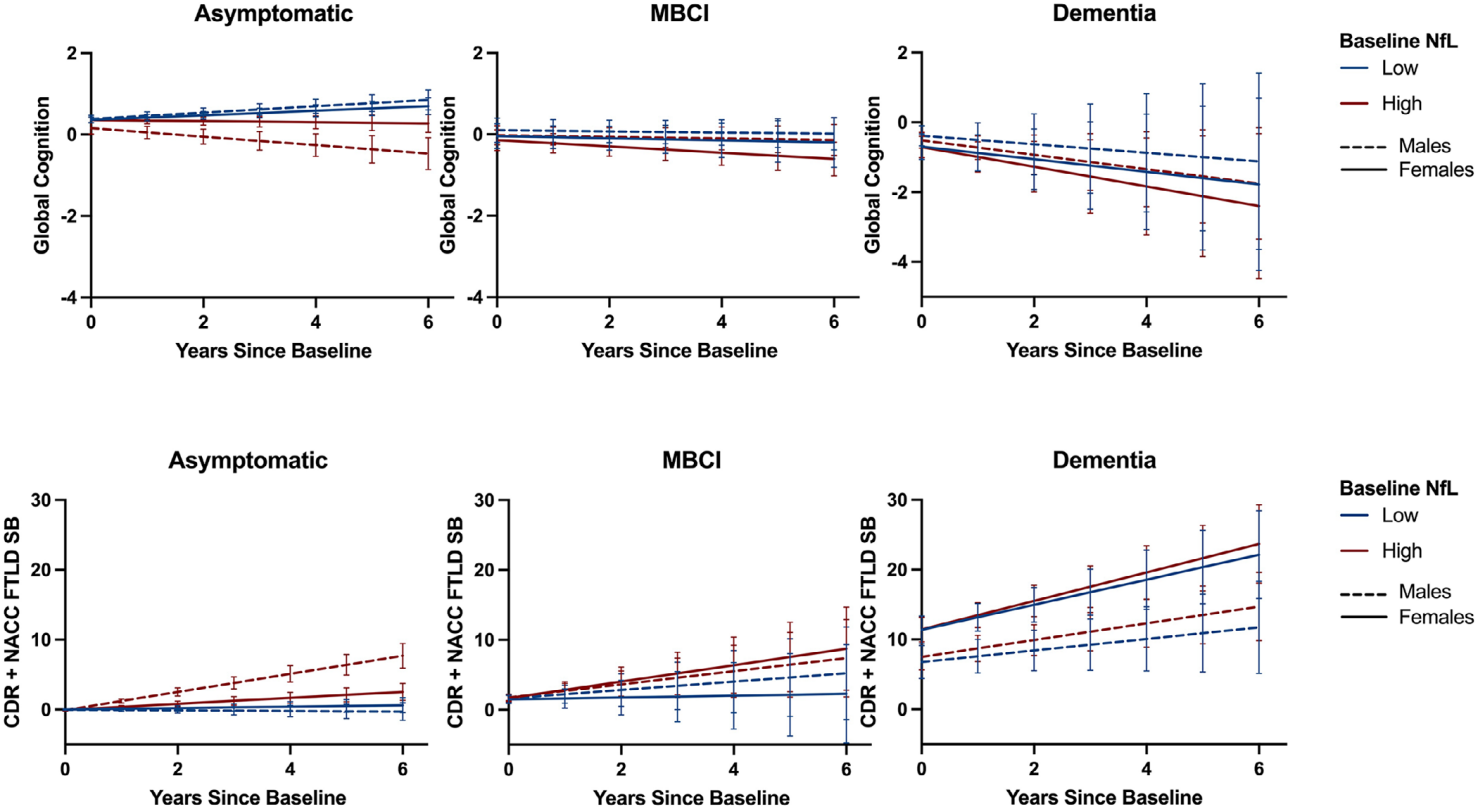
Female FTD pathogenic variant carriers exhibit better cognitive and functional trajectories for their baseline NfL concentration compared to males in asymptomatic stages only. Predicted plots based on linear mixed effects models, bars represent 95% CI. Predicted slopes estimated at low and high ranges (25th and 75th percentile) for continuous variables (e.g., baseline NfL levels). CDR + NACC FTLD, Clinical Dementia Rating dementia staging instrument plus National Alzheimer's Coordinating Center behavior and language domains frontotemporal lobar degeneration module; FTD, frontotemporal dementia; MBCI, mild behavioral/cognitive impairment; NfL, neurofilament light chain.

In our final series of models, we sought to understand differences in the association between NfL and clinical trajectories based on sex and disease stage (asymptomatic, MBCI, dementia). In models stratified by disease stage at study entry, there was an interaction among sex, baseline NfL, and time on cognition and functional trajectories over time, with the greatest sex differences in asymptomatic participants (Table 3). Among asymptomatic participants, females with higher baseline NfL concentrations exhibited less decline in cognition and function compared to males (Figure 2). Thus, the pattern of results suggested there were sex differences in the predictive utility of baseline NfL on clinical outcomes for asymptomatic participants. Models examining the predictive utility of baseline NfL did not demonstrate statistically significant sex differences on clinical outcomes among participants with MBCI or dementia at baseline.

**TABLE 3:** Models examining the predictive utility of baseline plasma neurofilament light chain (NfL) by sex on cognitive and functional trajectories, stratified by disease stage at study entry.

Outcome measure	Unstandardized effect size (standard error) for (baseline NfL x sex x time) parameter	*P*	95% CI
Global cognition			
Asymptomatic	0.01 (0.004)	0.005	[0.004, 0.020]
MBCI	−0.01 (0.008)	0.513	[–0.020, 0.010]
Dementia	−0.00 (0.015)	0.900	[–0.032, 0.028]
CDR + NACC FTLD SB			
Asymptomatic	−0.12 (0.020)	0.000	[–0.156, –0.079]
MBCI	0.06 (0.035)	0.100	[–0.011, 0.126]
Dementia	−0.02 (0.028)	0.573	[–0.071, 0.039]

Abbreviations: CDR + NACC FTLD SB,  CDR dementia staging instrument plus National Alzheimer's Coordinating Center behavior and language domains frontotemporal lobar degeneration module sum of boxes; CI, confidence interval; MBCI, mild behavioral/cognitive impairment.

Sensitivity analyses were again conducted controlling for informant sex. In disease‐stratified models examining the interaction among sex, baseline NfL, and time on cognitive trajectories, effects were attenuated, but of similar size and directionality. In contrast, the interaction effects were strengthened when examining functional trajectories in disease‐stratified models.

### 3.4 Post hoc analyses

#### 3.4.1 Genotype‐specific effects

To determine whether the observed sex differences differed by genotype, we conducted post hoc analyses stratifying final models by genotype. Our results suggested a similar pattern to the main findings, particularly in *GRN* pathogenic variant carriers (Table S3; Figure S1 in supporting information). Female *GRN* variant carriers with MBCI or dementia at baseline exhibited faster rates of cognitive and functional decline than males. A similar pattern of greater cognitive decline in females than males was observed in female *C9orf72* variant carriers with dementia; functional decline was similar across females and males with dementia. In *MAPT* carriers, females with dementia exhibited a more rapid functional decline (*P* < 0.001) and increase in plasma NfL (*P* = 0.001) than males with dementia across the study course. However, an opposite effect was observed for cognitive outcomes in *MAPT* variant carriers, such that females with dementia exhibited *less* cognitive decline than males with dementia (*P* < 0.001).

#### 3.4.2 NfL as a prognostic marker across autosomal dominant forms of FTD

While stratification based on sex, disease stage, and genotype is important to understand group‐specific effects and support precision medicine approaches, it is also clinically beneficial to examine whether NfL serves as a useful prognostic marker across participants with forms of autosomal dominant FTD. Post hoc analyses support NfL as a prognostic biomarker of clinical progression in this cohort; in line with previous reports, higher baseline NfL was associated with faster functional decline over time when combined across all participants (*b* = 0.07, *P* < 0.001, 95% CI [0.06, 0.08]).

## 4 DISCUSSION

Our data show important sex differences in the clinical manifestation of genetic FTD. Overall, females carrying pathogenic FTD variants in *MAPT*, *GRN*, or *C9orf72* presented with higher baseline plasma NfL concentrations and faster longitudinal increases of plasma NfL, an indicator of neuroaxonal degeneration, compared to males—particularly in later stages of disease. In the asymptomatic stages, females and males did not differ on cognitive and functional outcomes, and in fact, females exhibited *better* clinical trajectories for their pathological burden proxied by plasma NfL. However, after symptom onset, females with MBCI or dementia exhibited disproportionately steeper cognitive and functional declines compared to men. This pattern is consistent with cognitive resilience characterized by disproportionately delayed symptom manifestation for pathology burden in early stages of disease that may be followed by more rapid subsequent decline in female pathogenic variant carriers once they become symptomatic. These findings have several important clinical implications: (1) in early stages of disease, plasma NfL may not be as sensitive a prognosticator in females compared to males at risk for FTD, though this sex difference appeared to attenuate at later disease stages; (2) identification of biological pathways supporting female “resilience” may be high‐yield targets for FTD prevention; (3) after symptoms manifest, females may require more frequent monitoring to support a more rapid clinical trajectory; and (4) clinical trials in FTD should consider sex‐specific biological targets and monitoring techniques.

Our data are consistent with a pattern of “cognitive resilience” in females carrying pathogenic variants of FTD compared to men. Namely, females showed better functioning than males given a similar level of neurodegeneration in prodromal/asymptomatic stages, followed by a period of more precipitous decline after disease manifestation. Other traditional “cognitive resilience” factors, such as high educational attainment, are commonly associated with a pattern of preserved functioning until a point, at which the rate of decline can be subsequently much steeper presumably related to higher accumulated pathology burden.^27^ Indeed, a prior study demonstrated a cognitive resilience phenotype in females with bvFTD who exhibited better than expected executive and psychiatric functions based on their level of magnetic resonance imaging–based atrophy.^12^ We expand on this work suggesting that this pattern is present in FTLD pathogenic carriers and may be present in FTD more broadly (vs. limited to bvFTD). A very similar pattern of early outperformance followed by steeper clinical decline is also observed in females with AD.^2^, ^3^ This suggests there may be female‐specific biological pathways modulating manifestation of neurodegenerative diseases across etiologies, though opposite effects have also been described.^28^ Prior work has also demonstrated greater baseline empathy and prosocial behavior in females compared to males,^29^ which may uniquely contribute to female resilience against FTD behavioral and functional decline. This could be particularly relevant in our symptomatic sample, which consists primarily of individuals with dementia due to bvFTD and MBCI with behavioral features.

Several alternative interpretations also exist. One possibility is that our current diagnostic criteria set of cognitive, behavioral, and functional assessments, and the collateral report of male caregivers/spouses is less sensitive to the early signs and symptoms of FTD in females leading to a later age at initial diagnosis compared to males. Multiple studies have identified an earlier age of onset in males with FTD^10^, ^11^ and sex differences in initial symptom presentation,^30^ which may be consistent with a gender (versus sex) bias hypothesis. It is highly likely that social and cultural standards of gender roles influence how FTD is diagnosed and treated and cannot be teased apart from our study design. Nonetheless, there are two findings that are more consistent with more fundamental sex differences in FTD pathophysiology (versus gender). First, we found significantly steeper NfL slopes in female mutation carriers compared to males *regardless of clinical diagnosis* (asymptomatic to symptomatic). And second, we observed an *interaction* between sex and NfL showing attenuated cognitive and functional trajectories in asymptomatic females versus males; this suggests that for the same absolute level of NfL, females are showing disproportionately slower clinical progression (i.e., responding to disease biology differently) and should not be accounted for by gender‐biased diagnostics. Further, our sensitivity analyses controlling for the sex of the study informant in our analyses did not significantly alter the primary findings, suggesting that the observed relationships are detectable beyond potential gender bias in symptom reporting. Whereas the majority of our cohort consists of individuals with behavioral symptoms, females who present with a primary progressive aphasia often exhibit worse cognitive decline compared to males overall.^11^ Yet, existing studies are not stratified by disease stage limiting our understanding of sex differences in the course of disease progression. Future work is needed to wholistically evaluate the intersection of gender and biological sex to more comprehensively understand these findings.

Another implication of high clinical importance is that plasma NfL is a less sensitive predictor of impending cognitive and functional decline in females than males in early disease stages (Figure 2). Although sex differences in NfL concentrations have been examined cross‐sectionally in AD,^9^ additional work on longitudinal NfL trajectories is needed. Our findings contrast with those in AD in which tau and amyloid biomarkers in blood, CSF, or positron emission tomography track *more strongly* with clinical decline in females compared to men, particularly during early disease stages.^31^, ^32^, ^33^ Therefore, while some sex‐specific effects may be evident across neurodegenerative disorders, biomarker sensitivity and prognostic value may differ based on the protein measured and disease etiology. These sex‐specific clinical trajectories and biomarker utilities are highly relevant for prognostic counseling with families and in planning clinical trials. These data increasingly support development of precision dementia care models that account for biological sex.

The biological mechanisms underlying these sex differences remain unclear. Sex‐specific pathways of neuroimmune processes,^34^ neuro‐endocrine/hormonal changes,^35^ and X chromosome biology^36^ are emerging as candidate targets for tauopathies. For instance, females generally show more robust immune responses compared to males^37^ and in AD, microglial activation was shown to statistically mediate the relationship between amyloid beta and tau accumulation in females but not males.^34^ Dysregulation of neuroimmune processes is increasingly implicated in FTD.^38^, ^39^ It is possible that female gene carriers with FTD also show divergence in how immune functioning predisposes to risk of tau and TAR DNA‐binding protein 43 accumulation. Additionally, given that midlife onset of FTD coincides with the average age of menopause transition (age 51 in United States), sex hormone changes in females may be a particularly salient pathway to investigate. Changes in estrogen and progesterone may directly or indirectly impact protein accumulation^40^, ^41^, ^42^ and/or glymphatic clearance of neurotoxins.^35^ Finally, emerging evidence highlights the protective nature of the X chromosome in reducing mortality and preserving cognition in animal models of aging and AD.^36^ These effects may be driven by candidate genes, such as *Kdm6a*, that do not undergo X‐linked inactivation.^43^ Further querying of X chromosome biology in FTD is an important future area of work. Indeed, these converging data strongly suggest that females and males show fundamental differences in how neurodegenerative disease unfolds. Additional work is urgently needed to understand the mechanisms driving sex‐specific disease pathways in FTD.

In post hoc analyses, the pattern of our results appeared generally similar across genetic variants with steeper cognitive and functional decline in symptomatic females compared to males. Notably, there appeared to be a discrepant trend in *MAPT* variant carriers for cognitive outcomes only, such that *symptomatic males* exhibited faster rates of cognitive decline than females, despite showing similarly attenuated rates of functional decline. Prior work by some of our authors highlights the decreased utility of NfL as a prognostic biomarker in *MAPT* variant carriers compared to *GRN* and *C9orf72* variant carriers. Future work is needed to more precisely estimate the differential effects of sex, disease stage, and NfL concentrations on clinical trajectories among genetic variants of FTD, particularly given the small sample size of participants within each of these stratified groups. Additional consideration of the way in which gender‐related environmental and social factors impact and interact with sex differences is warranted.

There are several notable limitations of the current analyses, including lack of pathological data, small sample size of participants with MBCI/dementia in genotype‐stratified analyses, and lack of ethnoracial and socioeconomic diversity of the ALLFTD cohort (> 90% White participants), which may limit scope and generalizability of identified effects. Factors such as ethnicity, race, and geographical location contribute to comorbid vascular pathology, a health variable that disproportionally impacts cognition in post‐menopausal females compared to males^44^ and may further interact with disease progression. More broadly, recruitment bias in these cohorts may lead to underdiagnosis in groups with less access to research participation and medical care. This study focused on autosomal dominant FTD and it is unclear whether our findings generalize to the greater FTD population (e.g., sporadic cases). Finally, we focused on cognitive and functional trajectories and did not systematically examine sex differences in behavioral symptoms in FTD. This is the focus of a manuscript recently published by several of our co‐authors (Liu et al., 2025, in press)^45^.

## 5 CONCLUSIONS

Our study reveals sex differences in the progression of autosomal dominant FTD. We observed that females initially function better than expected based on their level of NfL, but subsequently experience an accelerated decline in cognitive and functional abilities during later stages of the disease. This pattern may indicate “resilience” against neurodegeneration in females, but it also highlights the limited predictive value of NfL in the early stages of FTD for this group. In contrast, NfL appears to be a more sensitive indicator of disease progression in males.

Moreover, the disease stage itself may provide a more accurate prognostic tool for females, helping clinicians better predict and manage the expected clinical trajectory. These findings contribute to the increasing body of evidence suggesting that neurodegenerative diseases manifest differently across sexes. Historically, women's brain health has received less attention^46^ and newly available treatments for other forms of dementia (e.g., AD) have shown less efficacy in females.^47^ As development of disease‐modifying treatments for FTD advance, it is crucial to conduct further research to understand the influence of sex‐specific biologic factors on FTD risk and progression.

## CONFLICT OF INTEREST STATEMENT

Dr. Litvan is a member of the Scientific Advisory Board for the Rossy PSP Program at the University of Toronto, Aprinoia, Amydis and the Food and Drug Administration (FDA) Peripheral and Central Nervous System Drugs Advisory Committee. She receives her salary from the University of California San Diego and as Chief Editor of *Frontiers in Neurology*. Dr. Staffaroni receives support from NIH/NIA, AFTD, Bluefield Project to Cure FTD. He is a member of the ADDF Scientific Review Board. Dr. Pantelyat is a member of a board at Ono Pharma and MedRhythms, Inc. Dr. Apostolova participates on boards at IQVIA, NIA, UAB Nathan Schock Center, New Mexico Exploratory ADRC, and FDA. Dr. Apostolova holds leadership positions at Medical Science Council Alzheimer's Association Greater IN Chapter, Alzheimer's Association Science Program Committee, FDA PCNS Advisory Committee, and Beeson Program Committee. Dr. Cassaletto participates on data safety and monitoring boards for Erlandson & Webel (HEALTH) and Wheeler (HEALTH‐Cog). She also serves in leadership roles for the International Neuropsychological Society and ISTAART Cognition PIA Steering Committee. Dr. Tartaglia serves as a scientific advisor for the Women's Brain Foundation, Brain Injury Canada, and PSP Canada. Dr. Illán Gala serves on scientific advisory boards at UCB and Nutricia. Dr. Rademakers serves on the scientific advisory board of Arkuda Therapeutics. Dr. Boeve serves on advisory boards for the Tau Consortium, AFTD, and LBDA. Dr. Petrucelli is chief scientific advisor for Target ALS and on the editorial board of *Science Translational Medicine*. Dr. Masdeu serves on an advisory board for Coya Therapeutics Inc. Drs. Memel, Garcia Castro, Tartaglia, Kornak, Saloner, Paolillo, Cadwallader, Gorno‐Tempini, Mandelli, Apostolova, Graff‐Radford, Bayram, Pressman, Miyagawa, Mackenzie, Darby, Appleby, Gendron, Heuer, Forsberg, Rojas, Brushaber, Domoto‐Reilly, Ghoshal, Lapid, Pascual, Lee, Marisa Ramos, Ramanan, Rascovsky, Snyder, Boxer, and Rosen do not have any conflicts to disclose. Anna VandeBunte, BA, Coty Chen, BS, Jill Goldman, MS, MPhil, and Nellie Brushaber, BS do not have any conflicts to disclose. Author disclosures are available in the supporting information.

## CONSENT STATEMENT

All participants provided written informed consent, and the study was approved by local institutional review boards.

## Supporting information



Supporting Information

Supporting Information
