# Correction to "Sex differences in the clinical manifestation of autosomal dominant frontotemporal dementia"

**DOI:** 10.1002/alz.70757

**Published:** 2025-09-23

Memel M, Staffaroni AM, Ilan‐Gala I, et al. Sex differences in the clinical manifestation of autosomal dominant frontotemporal dementia. *Alzheimers Dement*. 2025;21(4):e14630. https://doi.org/10.1002/alz.14630

The name of the third author is incorrect. “Ilan‐Gala I” should be replaced by Illan‐Gala I.

We apologize for this error.

